# Development and Evaluation of a Dual-Target One-Step Nested PCR for the Detection of Spotted Fever Group *Rickettsia* spp. in Ticks

**DOI:** 10.3390/pathogens15030312

**Published:** 2026-03-13

**Authors:** Phiaw Chong Foo, Canedy Jacob, Christina Injan Mawang, Ernieenor Faraliana Che Lah, Mariana Ahamad

**Affiliations:** Acarology Unit, Infectious Disease Research Centre, Institute for Medical Research, Ministry of Health Malaysia, National Institutes of Health Complex, Bandar Setia Alam, Shah Alam 40170, Selangor, Malaysia; canedy@moh.gov.my (C.J.); christinainjan@moh.gov.my (C.I.M.); erniee@moh.gov.my (E.F.C.L.);

**Keywords:** spotted fever *Rickettsia*, one-step nested PCR, tick-borne bacteria, molecular diagnosis, assay development

## Abstract

Spotted fever group (SFG) rickettsioses are tick-borne infectious diseases caused by more than 30 *Rickettsia* species. As ticks may harbor and transmit multiple pathogens during a single blood meal, sensitive and specific molecular detection methods are essential for early diagnosis. Conventional nested PCR is commonly used but is time-consuming and prone to cross-contamination due to multiple amplification steps. This study evaluated a dual-target one-step nested PCR assay developed as a rapid alternative to conventional nested PCR for SFG *Rickettsia* detection. Gene-specific primers targeting the *Rickettsia* outer membrane protein A (*ompA*) gene and the 17 kDa antigen gene were designed, with a *Plasmodium falciparum* thrombospondin-related anonymous protein (*TRAP*) gene included as an internal amplification control. Primer specificity was verified in silico, and assay performance was assessed using synthetic DNA templates. The dual-target one-step nested PCR achieved detection limits of 10 gene copies for the 17 kDa gene and 1000 gene copies for *ompA*, compared with 10 and 100,000 gene copies, respectively, using conventional nested PCR. Screening of 184 tick specimens identified one positive sample (0.54%) for the *Rickettsia* 17 kDa gene. Overall, the dual-target one-step nested PCR demonstrated comparable sensitivity to conventional nested PCR while reducing assay time and contamination risk, indicating its potential as a reliable tool for SFG *Rickettsia* detection.

## 1. Introduction

Ticks are among the most important arthropod vectors of zoonotic pathogens and are unique in their ability to transmit a diverse array of pathogens (including bacteria, viruses, and protozoa), often through long-term attachment and transovarial transmission [1,2]. Ticks are capable of harboring and transmitting multiple pathogens concurrently, making them of considerable medical importance due to their role as vectors. A single tick can harbor more than one pathogen, and co-infections have been reported in ticks and their vertebrate hosts. For example, *Ixodes ricinus* has been identified as a vector for *Borrelia* spp. and *Rickettsia* spp., which are causative agents of Lyme borreliosis and spotted fever group (SFG) rickettsioses, respectively [3,4].

The risk of humans acquiring tick-borne diseases has increased due to greater exposure to environments where ticks are commonly found. This pattern is influenced by ecological and environmental changes, as well as the presence and abundance of vertebrate hosts that maintain and amplify tick populations and their associated pathogens [5]. These infections often begin with general symptoms, such as fever, fatigue, or body aches, which can make early diagnosis more difficult [6,7]. It is therefore important to identify the specific pathogen involved, such as *Ehrlichia*, *Rickettsia*, or *Anaplasma*, because each one may require a different treatment approach, usually involving the use of doxycycline [5,6]. Failure to diagnose correctly or in a timely manner can lead to poor treatment outcomes and an increased risk of complications [5,7]. Since lab confirmation may take time, healthcare providers are encouraged to begin treatment when the symptoms and history of possible tick exposure suggest infection. This early response can help to reduce the risk of severe illness, but starting antibiotics without a confirmed diagnosis may result in unnecessary medication use or complicate later efforts to verify the exact cause.

Bacteria belonging to the genus *Rickettsia* are important tick-associated pathogens that pose considerable health risks to both humans and animals. These infections are primarily transmitted through tick bites and can cause a broad range of illness, from mild febrile conditions to severe systemic disease [8]. Advancements in whole-genome sequencing have led to the classification of *Rickettsia* into four major phylogenetic clades: the ancestral group (AG), the spotted fever group (SFG), the typhus group (TG), and the transitional group (TRG) [9,10]. Each group differs in terms of evolutionary lineage, vector associations, and pathogenic potential. Recognizing these groupings is crucial for improving diagnostic precision, enhancing disease surveillance, and informing vector control strategies, especially in regions experiencing the emergence or resurgence of tick-borne diseases.

Among these groups, the spotted fever group holds particular clinical relevance due to its involvement in acute febrile illnesses in humans [11]. Transmission can occur rapidly following a tick bite as the bacteria efficiently invade vascular endothelial cells and initiate a systemic inflammatory response [12]. The common clinical manifestations include high fever, headache, and a variable rash. In more severe or untreated cases, the disease may progress to complications involving the central nervous and cardiovascular systems, driven by extensive endothelial damage [13]. A clear understanding of these clinical patterns and their pathogenesis is vital for early recognition and effective treatment of infections caused by SFG rickettsiae.

Serological testing remains a cornerstone for the diagnosis of tick-borne diseases [14]. The current gold-standard serological assays include the enzyme-linked immunosorbent assay (ELISA) and Western blot, often visualized using the indirect immunofluorescence assay (IFA) [15]. Recent advances in immunoblot-based platforms, including line immunoblot assays, have improved antigen selection and interpretative frameworks for several tick-borne infections, such as relapsing fever borreliosis and Lyme disease [16,17]. Nevertheless, despite these developments, serological methods remain limited in the early phase of infection due to delayed antibody responses and potential cross-reactivity among closely related pathogens. Indeed, fewer than 40% of early cases may be correctly identified by ELISA, and false-positive rates of up to 28% have been reported for Western blot assays [18,19]. In addition, serological testing typically requires several days to generate results, which can delay clinical decision-making. Consequently, molecular techniques such as conventional PCR, nested PCR, and real-time PCR are increasingly preferred for the early and direct detection of *Rickettsia* DNA in vectors and clinical specimens, where timely and sensitive identification is critical for surveillance and diagnostic applications [14,15,18,19].

In Malaysia and other Southeast Asian countries, several studies have reported the presence of spotted fever group (SFG) *Rickettsia* in ticks, wildlife, and domestic animals, indicating a persistent risk for human exposure [8,9,10,20,21,22]. Despite this documented presence, routine molecular screening of ticks is not yet standardized in Malaysia, and the available assays remain largely research-based. Molecular techniques such as conventional PCR, nested PCR, and real-time PCR are preferred for *Rickettsia* detection because they offer higher sensitivity during the early stages of infection when compared with serological assays [14,15,18,19]. However, these molecular methods present practical challenges for routine surveillance; conventional nested PCR requires two separate amplification rounds, which increases turnaround time and the risk of laboratory contamination [23]. Conversely, while real-time PCR is highly effective, it requires specialized instrumentation and incurs higher operational costs, making it less accessible for resource-limited settings [15].

The present study aimed to develop an in-house single-step nested PCR assay that is capable of simultaneously amplifying two *Rickettsia* genes. Additionally, an exogenous internal amplification control (IAC) based on the *Plasmodium falciparum* thrombospondin-related anonymous protein (*TRAP*) gene was incorporated to monitor PCR inhibition and distinguish true-negative results from amplification failure [23,24]. Subsequently, the optimized thermostabilized PCR assay was evaluated for its sensitivity and specificity and further validated using tick DNA specimens to detect the presence of *Rickettsia* spp.

This study provides a methodological improvement for molecular detection of SFG *Rickettsia* by introducing a dual-target one-step nested PCR assay incorporating an internal amplification control. The assay is designed to reduce hands-on time, minimize contamination risk, and enhance diagnostic reliability, particularly in laboratories with limited resources. Such improvements are important for strengthening tick-borne disease surveillance and supporting early pathogen detection.

## 2. Materials and Methods

### 2.1. Reagents and Apparatus

All oligonucleotides were synthesized by Integrated DNA Technologies (Singapore). PCR reagent components, including recombinant *Taq* DNA polymerase, 25 mM dNTP Mix, 2.5 mM MgCl_2_, 1× *Taq* buffer with (NH_4_)_2_SO_4_ and ultra-pure DNase/RNase-Free distilled water, were purchased from Thermo Fisher Scientific, Waltham, MA, USA. Agarose powder for gel electrophoresis was purchased from Next Gene Scientific, Malaysia (OEM from Lonza Seakem LE Agarose, Rockland, MA, USA), while Sample Loading dye, Tris-Borate-EDTA buffer, 50 bp DNA ladder and DNA gel stain were purchased from Thermo Fisher Scientific, Waltham, MA, USA. All the PCR reactions were carried out using Mastercycler Pro S vapo.protect (Eppendorf, Hamburg, Germany). The PCR amplicons were analyzed using Owl EasyCast Horizontal Gel Electrophoresis System (Thermo Fisher Scientific, Waltham, MA, USA) and visualized using UVP ChemStudio Series (Analytik Jena, Jena, Germany). All the samples were extracted using QIAamp DNA Mini Kit (Qiagen, Hilden, Germany).

### 2.2. Primer Design

**Figure 1:**
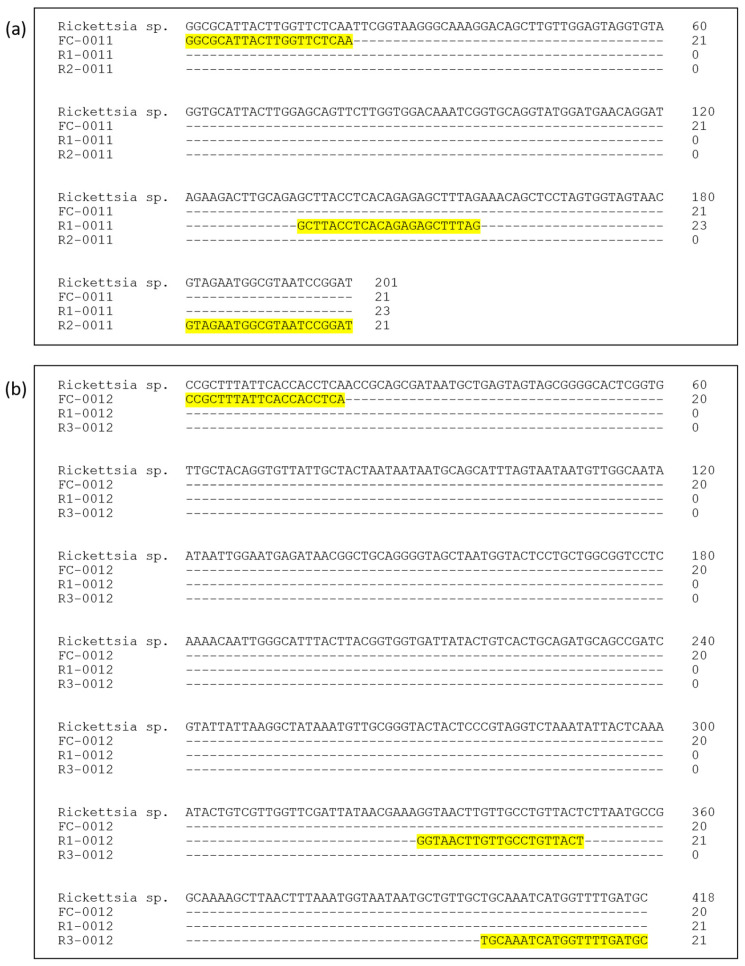
Primer regions on (**a**) 17 kDa gene sequence and (**b**) *ompA* gene sequence. Yellow highlights indicate the binding positions of the outer and inner primers used in the dual-target one-step nested PCR assay.

Two *Rickettsia* gene targets, the 17 kDa surface antigen (17 kDa) gene and the outer membrane protein A (*ompA*) gene, were selected to provide dual-target detection as both genes are widely used molecular markers for spotted fever group *Rickettsia* and offer complementary advantages in sensitivity and specificity. The primer sets used were designed based on the conserved region of the reported *ompA* gene (GenBank accession nos. KF691749.1, KJ735645.1 and KX158267.1) and 17 kDa gene (GenBank accession nos. KC107823.1, U17008.1 and AY281069.1). For each target gene, three primers were designed: one common forward primer, one outer reverse primer, and one inner reverse primer. This configuration enables nested amplification to occur within a single reaction tube, thereby enhancing sensitivity while avoiding a second amplification step. The binding locations of primers are shown in Figure 1. These primers were tested in silico and empirically before being incorporated into PCR mix for development of one-step nested PCR.

The primers used to target *Rickettsia* 17 kDa gene were named as FC-0011, R1-0011 and R2-0011, while FC-0012, R1-0012 and R3-0012 were designed to target *Rickettsia ompA* gene. FC-0011 was used as common forward primer that can form amplicon with size 201 bp when complemented with outer reverse primer R2-0011, and 157 bp when complemented with inner reverse primer R1-0011. Meanwhile, FC-0012 was used as common forward primer that can form amplicon with size 418 bp when complemented with outer reverse primer R3-0012 and 350 bp when complemented with inner reverse primer R1-0012.

The primer set working as internal amplification control (IAC) in dual-target one-step nested PCR was designed using the *Plasmodium falciparum* thrombospondin-related anonymous protein (*TRAP*) gene sequence (GenBank accession no. ON332429.1). The *Plasmodium falciparum TRAP* gene was selected as an IAC because it is a non-target exogenous sequence with no known homology to tick or rickettsial genomes. This ensures that the IAC monitors for PCR inhibitors without competing with target templates or producing non-specific amplification. Amplification of IAC uses primer pair F-IC-0014 and R-IC-0014, which generated amplicon with size 268 bp. All three primer sets were designed using PrimerQuest Tool software, which is available from the Integrated DNA Technologies (IDT) website [https://sg.idtdna.com (accessed on 15 April 2021)]. All the sequences of primers used in this study are listed in Table 1.

### 2.3. Formulation of Dual-Target One-Step Nested PCR

The final concentrations of primers used were 1.2 µM FC-0011, 0.5 µM R1-0011, 0.2 µM R2-0011, 1.5 µM FC-0012, 1 µM R1-0012, 0.5 µM R3-0012, 0.25 µM F-IC-0014 and 0.25 µM R-IC-0014. The amplification was carried out in a final volume of 20 μL containing 1 × PCR buffer, 2.5 mM of MgCl2, 0.44 mM of dNTPs mix, 3.75 U of *Taq* DNA polymerase, 1pg of synthetic *TRAP* gene DNA and 2 μL of target DNA template. PCR reaction was performed with initial denaturation at 95 °C for 5 min, followed by 40 cycles of 95 °C for 30 s, 60.5 °C for 30 s and 72 °C for 30 s; and a final extension at 72 °C for 5 min. The product was subjected to gel electrophoresis in 2% *w*/*v* agarose gel stained with SYBR Safe DNA gel stain, electrophoretically run under 90 V for 90 min and visualized using Gel Documentation System (UVP ChemStudio Series, Analytik Jena).

### 2.4. Specimen Sample Testing and Data Analysis

A total of 184 tick specimens of various species, previously morphologically and molecularly identified, were obtained from earlier studies [20,25]. These included various vegetation tick species collected from forest areas in the states of Pahang and Terengganu in 2018 and 2019. Prior to DNA extraction, ticks were first cleaned with hydrochloric acid (HCl) solution, then immersed in 70% ethanol to neutralize any residual acid, followed by two rinses of distilled water to remove traces of ethanol and finally macerated manually using a sterile scalpel, after which 80 µL of phosphate-buffered saline (PBS) was added. DNA extraction was subsequently carried out using the QIAamp DNA Mini Kit, following the manufacturer’s protocol with slight modifications as described by Ernieenor et al. [25]. Total genomic DNA extracted from each of the 184 individual ticks was screened for the presence of *Rickettsia* spp. using the developed dual-target single-step nested PCR assay. Any amplified PCR product was sequenced using First BASE DNA sequencing service (First BASE Laboratories, Seri Kembangan, Malaysia). The obtained sequence was subsequently analyzed using the BLASTn (BLAST+ 2.11.0) algorithm via the National Center for Biotechnology Information (NCBI) database.

## 3. Results and Discussion

### 3.1. Development of Dual-Target One-Step Nested PCR

**Figure 2:**
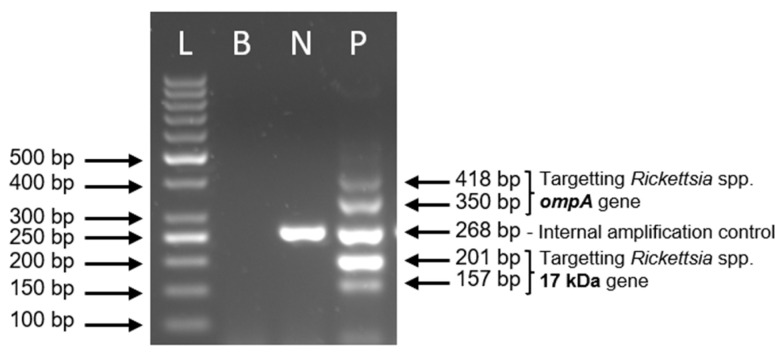
Representative agarose gel electrophoresis of the developed dual-target one-step nested PCR. L, 50 bp DNA ladder; B, blank control; N, valid negative control; P, positive control.

The dual-target single-step nested PCR assay was optimized and standardized using synthetic DNA constructs based on the 17 kDa and *ompA* genes. The outcome of the optimization (gel electrophoresis) is presented in Figure 2. Optimization began by determining the optimal primer ratio among three primer sets, with the aim of producing five amplicons exhibiting comparable band intensity on agarose gel electrophoresis. Following multiple rounds of empirical testing, the optimum primer concentrations were established as follows: 1.2 µM FC-0011, 0.5 µM R1-0011, 0.2 µM R2-0011, 1.5 µM FC-0012, 1 µM R1-0012, 0.5 µM R3-0012, 0.25 µM F-IC-0014 and 0.25 µM R-IC-0014. Further optimization identified that 0.44 mM of dNTP mix and 3.75 U of *Taq* DNA polymerase were reliably sufficient to generate all five amplicons in a single amplification reaction.

Three short synthetic DNA fragments corresponding to the 17 kDa, *ompA*, and *TRAP* genes were used as templates for the assay’s positive controls and internal amplification control (IAC). While the 17 kDa and *ompA* synthetic DNA samples were included exclusively in the positive control tubes, the *TRAP* gene was incorporated into the assay reagent mix prior to the addition of extracted DNA from ticks. The IAC was designed to validate the accuracy of negative results by confirming successful amplification of an IAC-specific amplicon on agarose gel after PCR, thereby excluding the possibility of false negatives due to PCR inhibition by co-extracted substances.

Incorporating the IAC in every reaction facilitates differentiation between true-negative and invalid results. A reaction was considered invalid if neither the target DNA nor the IAC amplicon was amplified, indicating potential inhibition. Conversely, the absence of the IAC amplicon in reactions yielding a positive result was acceptable as this may result from competition among primers when high concentrations of target DNA are present [26,27].

Nested PCR is widely recognized for its enhanced specificity compared to conventional single-primer PCR. However, traditional nested PCR involves two separate amplification runs, effectively doubling the reaction time and introducing additional pipetting steps, thereby increasing the risk of contamination. In contrast, the dual-target one-step nested PCR assay developed in this study requires only a single reaction run. All the primers needed for both the outer and inner amplification steps are incorporated into one tube and operate simultaneously. Figure 2 demonstrates the PCR product profiles for both negative and positive control reactions.

### 3.2. Performance of Dual-Target One-Step Nested PCR

**Figure 3:**
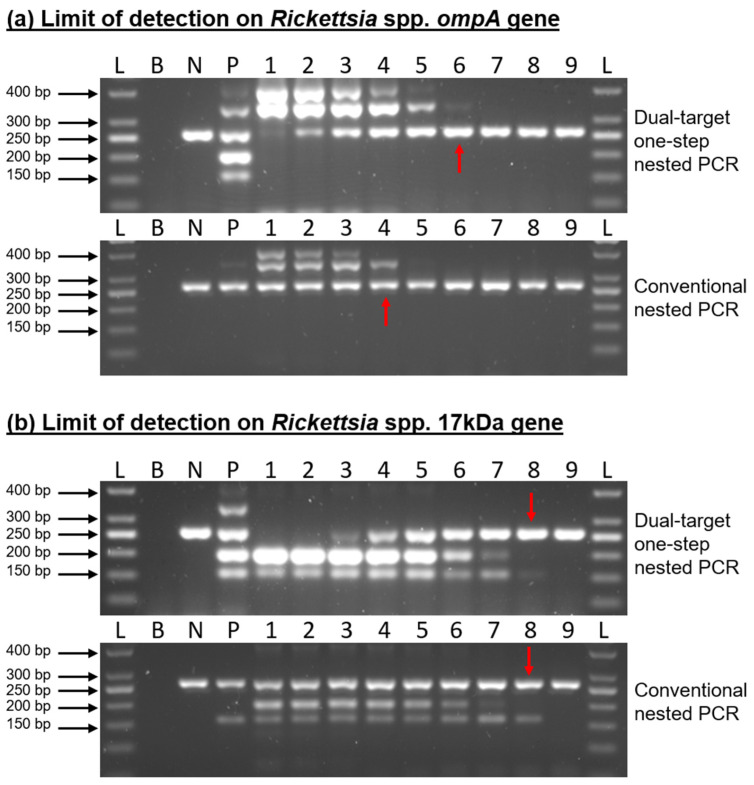
Detection limit comparison between dual-target one-step nested PCR and conventional nested PCR. L, 50 bp DNA ladder; B, blank control; N, valid negative control; P, positive control; 1, 1 × 10^8^ gene copies; 2, 1 × 10^7^ gene copies; 3, 1 × 10^6^ gene copies; 4, 1 × 10^5^ gene copies; 5, 1 × 10^4^ gene copies; 6, 1 × 10^3^ gene copies; 7, 100 gene copies; 8, 10 gene copies; 9, 1 gene copy. The red arrow indicates the lowest detectable dilution (detection limit) observed for the respective assay.

To evaluate the performance of the developed dual-target one-step nested PCR assay, an analytical sensitivity test was conducted to determine its detection limit. This evaluation was performed in parallel with the conventional nested PCR using the same primer sets and 10-fold serial dilutions of synthetic 17 kDa and *ompA* DNA templates, ranging from 1 × 10^8^ gene copies to 1 gene. The developed assay demonstrated a sensitivity comparable to that of the conventional nested PCR. For the detection of the *Rickettsia* spp. *ompA* gene, the detection limit of the developed assay was 1000 gene copies, while the conventional nested PCR exhibited a lower sensitivity, with a detection limit of 100,000 gene copies. In contrast, for the 17 kDa gene target, both the developed and conventional nested PCR assays demonstrated an identical detection limit of 10 gene copies (Figure 3). For this study, diagnostic sensitivity and specificity percentages were not calculated due to the limited number of positive field samples and the absence of a suitable gold-standard comparator. Therefore, assay performance was evaluated in terms of analytical sensitivity using defined DNA copy numbers.

Several molecular assays targeting the 17 kDa antigen gene have been reported for the detection of spotted fever group *Rickettsia* in ticks and clinical specimens. Previous studies have shown that nested PCR assays targeting the 17 kDa gene are capable of detecting low DNA copy numbers, often in the range of 10–100 gene copies, reflecting the highly conserved nature of this target [9,28]. In the present study, the dual-target one-step nested PCR demonstrated a detection limit of 10 gene copies for the 17 kDa gene. This is not only comparable to the sensitivity reported in earlier conventional nested PCR-based assays but also highlights that the transition to a one-step format does not compromise analytical sensitivity.

In contrast, the *ompA* gene is generally more challenging to amplify due to its larger size and sequence variability among SFG *Rickettsia* species [29]. The higher detection limit observed for the *ompA* target in this study is consistent with historical molecular assays targeting this gene. Despite this limitation, *ompA* remains valuable for confirmatory detection and species-level discrimination [8]. Notably, our assay outperformed the conventional nested PCR for this target, achieving a 100-fold lower detection limit (1000 copies vs. 100,000 copies), which represents a clear technical advantage.

A major advantage of the dual-target one-step nested PCR developed in this study over the conventional nested PCR protocols used in many regional studies is its closed-tube format. In standard nested PCR workflows, the requirement of two separate amplification runs necessitates opening reaction tubes between rounds, which substantially increases the risk of laboratory cross-contamination by aerosolized amplicons [23]. By integrating both the outer and inner primers into a single reaction, the present assay eliminates this step, thereby reducing contamination risk while also shortening overall processing time. Furthermore, the incorporation of the *TRAP* gene as an IAC provides an additional layer of quality assurance. Tick tissues are known to contain PCR inhibitors that may lead to false-negative results; therefore, confirmation of successful amplification through a positive IAC signal allows reliable discrimination between true-negative and invalid reactions [26,27]. This approach enhances diagnostic robustness compared with assays relying solely on external controls.

Real-time PCR (qPCR) assays are currently considered to be a standard approach for the detection of spotted fever group *Rickettsia* due to their rapid turnaround time and high analytical sensitivity [28]. However, qPCR requires specialized instrumentation, fluorescent probes, and dedicated laboratory infrastructure, which may limit its routine implementation in resource-limited or field-based surveillance laboratories [7]. In contrast, the dual-target one-step nested PCR developed in this study can be performed using conventional thermocyclers and standard agarose gel electrophoresis while maintaining high analytical sensitivity. Although the present study did not include a direct experimental comparison with qPCR, the simplified single-tube format, reduced contamination risk, and lower equipment requirements support the potential utility of this assay as a complementary tool for tick surveillance and research laboratories where real-time PCR platforms are not readily available.

### 3.3. Specimen Sample Testing

**Figure 4:**
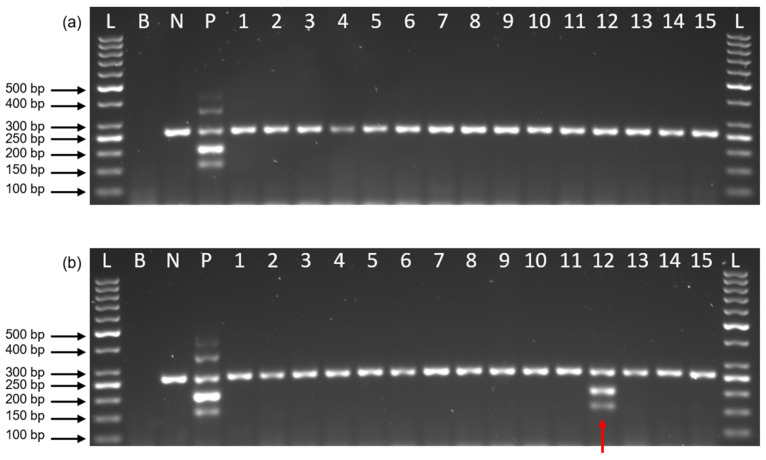
Representative agarose gel electrophoresis of the developed dual-target one-step nested PCR using tick DNA samples. (**a**) Gel image showing representative amplification results from tick samples that tested negative for *Rickettsia* spp.; (**b**) Gel image showing amplification results including one positive sample for the *Rickettsia* sp. 17 kDa gene (lane 12, indicated by the red arrow). L: 50 bp DNA ladder; B: blank control; N: valid negative control; P: positive control; lanes 1–15: representative tick DNA samples tested in the assay.

In this study, a total of 184 tick genomic DNA samples were screened for the presence of *Rickettsia* spp. Only one (0.54%) specimen was found to be positive for *Rickettsia* spp., which was isolated from the tick *Ixodes granulatus*, collected from Pahang, Malaysia. A sequence analysis of the 17 kDa gene revealed 100% similarity to *Rickettsia* sp. ATT (AF483196) (Table 2). Figure 4a illustrates an agarose gel image with no *Rickettsia* spp. gene detected, while Figure 4b presents a positive result for the *Rickettsia* sp. 17 kDa gene.

The low prevalence of 0.54% observed in this study is consistent with the focal and often sporadic distribution of *Rickettsia* spp. within tick populations in Malaysia and the wider Southeast Asian region, where pathogen presence is strongly influenced by ecological factors such as tick species, host availability, and habitat specificity [8,9]. Tick-borne rickettsiae are known to exhibit strong ecological associations with specific tick–host systems, which can result in low detection rates when sampling is geographically or temporally limited. The identification of *Rickettsia* sp. ATT in *Ixodes granulatus* is ecologically significant as *I. granulatus* has previously been identified as a potential vector of tick-borne pathogens in Peninsular Malaysia [25]. This association is consistent with regional metagenomic analyses that have characterized *I. granulatus* as harboring diverse bacterial communities, including potential zoonotic agents [20].

In this study, although the assay evaluation was limited by a small field sample size (n = 184) and low positivity (0.54%), the primary objectives remained methodological development and rigorous analytical validation. Standardized synthetic DNA templates were utilized to establish precise detection limits, ensuring a controlled comparison of the dual-target format against conventional nested PCR, independent of field prevalence. Furthermore, as the developed assay was designed for vector surveillance, validation was restricted to tick DNA samples only. Future research should evaluate clinical specimens (e.g., blood and tissue samples), cultured isolates, and larger geographically diverse field samples to assess the assay’s broader utility in clinical diagnostic settings.

## 4. Conclusions

In conclusion, the dual-target one-step nested PCR assay developed in this study represents a robust and practical molecular tool for the detection of SFG *Rickettsia*. By simultaneously targeting the 17 kDa and *ompA* genes within a single-tube format, the assay provides analytical sensitivity that meets or exceeds conventional nested PCR, particularly for the *ompA* target, while significantly reducing processing time and the risk of laboratory contamination. The integration of an IAC further ensures diagnostic reliability by ruling out false negatives caused by PCR inhibition, a critical feature for field-derived specimens. Ultimately, this streamlined approach offers a viable cost-effective alternative for routine surveillance and research laboratories, contributing to strengthened tick-borne disease monitoring and supporting early pathogen detection in endemic regions.

## Figures and Tables

**Table 1:** Oligonucleotide sequences used in this study.

Primers	Sequences (5′-3′)	Length (mer)	Product Size
**17 kDa surface antigen (17 kDa)**			
FC-0011	GGCGCATTACTTGGTTCTCAA	21	
R1-0011	CTAAAGCTCTCTGTGAGGTAAGC	23	157 bp
R2-0011	ATCCGGATTACGCCATTCTAC	21	201 bp
***Rickettsia* outer membrane protein A (*ompA*)**			
FC-0012	CCGCTTTATTCACCACCTCA	20	
R1-0012	AGTAACAGGCAACAAGTTACC	21	350 bp
R3-0012	GCATCAAAACCATGATTTGCA	21	418 bp
**Internal amplification control (IAC)**			
F-IC-0014	CAGCAAGTTGTGGTGTTTG	19	
R-IC-0014	GGTGAAGGTTCTTGTGGATTA	21	223 bp

**Table 2:** Tick specimen analysis—molecular detection and identification of *Rickettsia* spp. from 184 specimens.

Locality	Tick Species	Specimen Detected with *Rickettsia* spp.	Closest GenBank Identity [Gene: Accession Number]
Pahang	*Ixodes granulatus*	1/28	100% *Rickettsia* sp. ATT [17 kDa: AF483196]
	*Haemaphysalis* sp.	0/11	
	*Dermacentor* sp.	0/16	
	*Amblyomma* sp.	0/7	
	*Ixodes* sp.	0/2	
	*Dermacentor auratus*	0/2	
	*Dermacentor atrosignatus*	0/1	
Terengganu	*I. granulatus*	0/6	
	*Haemaphysalis* sp.	0/1	
	*Haemaphysalis hystricis*	0/1	
	*Dermacentor* sp.	0/29	
	*Amblyomma* sp.	0/1	
	*Amblyomma cordiferum*	0/2	
	*Ixodes* sp.	0/2	
	*D. auratus*	0/55	
	*D. atrosignatus*	0/9	
	*Dermacentor compactus*	0/1	
	*Dermacentor steini*	0/10	

## Data Availability

The data presented in this study are included in the article.
